# A case of ciprofloxacin-induced erythema multiforme

**DOI:** 10.4103/0253-7613.40490

**Published:** 2008

**Authors:** Santoshkumar R. Jeevanagi, S. Manjunath, V.K. Wali

**Affiliations:** Departments of Pharmacology, M.R. Medical College, Gulbarga, India; 1 Departments of Dermatology, M.R. Medical College, Gulbarga, India

**Keywords:** Ciprofloxacin, erythema multiforme

## Abstract

A 25 year old male admitted to the medical college hospital with presenting complaint of fever, skin rashes and itching. Before admission he had consulted a local doctor for fever, who had prescribed him Tablet ciprofloxacin 500mg twice daily and Tablet. Paracetamol 500 mg thrice daily. The patient had taken 6 tablets of ciprofloxacin before he developed the above symptoms. On detailed clinical examination multiple erythematous papule and plaques were present on the face, abdomen and neck. Target (Iris) lesions were seen on the extersor surface of both upper and lower limbs measuring about 1-2 cm in size. Multiple erythematous lesion were also found in mucosa of soft and hard palate, Uvula and posterior pharyngeal wall. Lower lips were swollen and edematous, Lymphnodes in the neck were enlarged and tender. The clinical features with which the patient presented were similar to those seen in a typical case of erythema multiforme and the patient recovered after stopping ciprofloxacin, Further rechallenge with oral ciprofloxacin was not done in the interest of patient and due to ethical constraints. This case is being reported for rare and potential fatal drug reaction with ciprofloxacin.

Ciprofloxacin is a fluorinated quinolone having broad antimicrobial activity and is effective after oral or parenteral administration. Side effects with the use of ciprofloxacin are relatively few and development of resistance by microbes is not rapid.[1] It is used in urinary tract infection, sexually transmitted diseases, and infections of the gut, respiratory tract, bones, and soft tissues. A few cases of ciprofloxacin-induced photosensitivity, hypersensitivity, anaphylaxis, vasculitis, erythema multiforme, Stevens-Johnson syndrome, or toxic epidermal necrolysis have been reported so far.[1–3] We report herewith a rare case of ciprofloxacin-induced erythema multiforme.

A 25-year-old male (weighing 55 kg) was admitted to Basaveshwar Teaching and General Hospital, Gulbarga, with complaints of fever, rashes, and itching. Fever, present for 6 days, was moderate in grade and was associated with diarrhea that was watery in nature, with no blood or mucus present in the stools, and without abdominal cramping.

The patient had consulted a doctor in his own locality and was prescribed ciprofloxacin (Ciplox™) tablets, 500 mg twice daily for 10 days, and paracetamol (Crocin™) tablets, 500 mg thrice daily for 5 days. He was also advised oral rehydration. After consuming six tablets of ciprofloxacin in 3 days, the patient noticed rashes and vesicles over his upper and lower limbs, which was associated with itching. The fever had not subsided, though the diarrhea was controlled. At this stage, he got admitted to the hospital. A detailed personal and family history of the patient was taken. He was a nonalcoholic and a nonsmoker. He had no history of allergy to paracetamol or any other drug. Various investigations like hemoglobin estimation, total and differential WBC count, ESR, routine urine examination, chest x-ray, blood urea, serum creatinine, and blood sugar were all within normal limits. Peripheral smear for malarial parasite, the Widal test, and HIV test were also negative. On dermatological examination multiple erythematous papules and plaques were present on the face, abdomen, and back. Target (iris) lesions were seen on the extensor surfaces of both the upper and lower limbs, measuring about 1-2 cm in size. On ENT examination, multiple erythematous lesions were present over the mucosa of the soft and hard palate, uvula, and posterior pharyngeal wall. His lower lips were swollen and edematous. Lymph nodes in the neck were enlarged and tender. The mucous membranes of the eye, nose, and genitalia were not involved.

The clinical features with which the patient presented were similar to those seen in a typical case of erythema multiforme[4] and the patient recovered after stopping ciprofloxacin. Conditions like herpes simplex, blistering skin diseases (e.g., pemphigus vulgaris and bullous pemphigoid), mucocutaneous diseases (e.g., Behcet's syndrome and Reiter's syndrome), and vasculitides (e.g., systemic lupus erythematosus and polyarteritis nodosa) were excluded clinically. We therfore concluded that this was a case of ciprofloxacin-induced erythema multiforme. Further rechallenge with oral ciprofloxacin was not done in the interest of the patient and due to ethical constraints. The patient was treated with injection ceftriaxone 1 gm IV twice daily, injection dexamethasone 4 mg IV thrice daily, tablet cetrizine 10 mg once daily, and tab paracetamol 500 mg thrice daily. He was discharged after 7 days when all clinical features of the disease had subsided.

The appearance of erythema multiforme in this patient who had taken oral ciprofloxacin could not be explained by any other concurrent disease or drug or chemical intake, and a dechallenge with ciprofloxacin improved the condition. This reaction is dose unrelated and can be labeled as a type B class of adverse effect.[5] It can also be considered as probable/likely as per causality assessment.[6]

With this case report we aim to create awareness about rare but potentially fatal drug reaction like erythema multiforme that can occur with ciprofloxacin which is one of the most commonly used antibacterial drugs in India.
